# A “Necessary Evil”: Staff Perspectives of Soft Restraint Kit Use in a High-Security Hospital

**DOI:** 10.3389/fpsyt.2020.00357

**Published:** 2020-05-14

**Authors:** Helen Walker, Lindsay Tulloch

**Affiliations:** ^1^Department of Health and Life Sciences, University of the West of Scotland, Paisley, United Kingdom; ^2^The State Hospital, Carstairs, Scotland

**Keywords:** mechanical restraints, emergency response belts, forensic mental health, soft restraint kit, high secure hospital, seclusion and restraint

## Abstract

**Introduction:**

Forensic mental health nurses working at the forefront of services can intermittently face enduring and somewhat harrowing or stressful situations. Enclosed is an example of the use of mechanical restraints (Soft Restraint Kit) for a two month period. Staff experience of working under such circumstances is an under reported area.

**Methods:**

The experience of nursing a patient under extreme conditions was captured through use of a qualitative study, using semi structured interviews with a purposive sample of (n = 10) staff nurses and nursing assistants in a high-security hospital.

**Results:**

Thematic analysis was undertaken generating four themes: sense of responsibility, aptitude, enablers/inhibitors, and consequence. Conclusions suggest that Soft Restraint Kits provide a useful method of containment, although prolonged use presents considerable challenges for staff. The importance of preparation and training cannot be underestimated and continued support and supervision are absolutely essential.

## Introduction

Nurses need to ensure they are actively involved in discussions relating to the management of violence, because staff safety is a primary concern in forensic mental health facilities (1). It is widely reported that high secure hospitals across the UK experience a high number of violent and aggressive incidents (2). Different strategies and approaches are used to deal with violent and threatening behaviour (3). In secure settings, physical, relational and procedural security measures are implemented in order that care can be safely delivered whilst at the same time risk can be managed (4). Due to the complexity of violence as a phenomenon it is perhaps best dealt with using a multi-professional approach (5).

Conflict and containment, for example, seclusion and manual restraint are important matters for hospital management and nursing practice (6). Extreme measures, such as those highlighted, are most commonly used when there is a risk of harm to others (7). It is the increasing emphasis on the use of restrictive practices that is a concern and seclusion is becoming a contentious practice (8). The challenge to clinicians, especially nurses, is to provide a safe environment while dealing with volatile patients and they may have little alternative to seclusion, having exhausted all other interventions (8). Nursing perspectives on the subject of reducing and eliminating containment methods, such as seclusion and mechanical restraint, have recently been captured through a large scale survey undertaken in Australia (9). Respondents viewed these containment methods as last resorts to maintain staff and patient safety.

Precipitants for seclusion are usually impending bodily harm to the patient or others and suicidal behaviour (5, 10); although NICE guidelines (2015) (11) never recommend seclusion for self harm or suicidal behaviour. The highest seclusion rates tend to be found in patients with diagnoses of psychosis, mania, personality disorder and intellectual disability (12).

A number of challenges emerge when patients demonstrate extreme violence repeatedly, especially when they require prolonged use of physical restraint. In such circumstances the use of mechanical constraints is usually considered. The rationale for this is linked to the risks to a person’s life from positional asphyxia. This can be brought on by many factors including a prolonged struggle and restraining of a person for extended periods of time in the prone (face down) position, especially if weight is applied to their back. People who are obese, on high-dose anti-psychotics or have pre-existing cardiac or respiratory conditions are particularly high risk in this position. Chan et al. (13) directly measured the restrictions on breathing during physical restraint and observed a 10% reduction in the supine (face up) position compared with a 15% reduction in the prone position. As a consequence of this risk to life, exploration regarding other restraint devices, which may allow a greater degree of control over prolonged violent persons, has been conducted within the research realm, and in the year 2000 led some organisations to adopt the use of a mechanical restraint called Emergency Response Belts (ERB’s). The name was changed to Soft Restraint Kits (SRK) around 2017, these two terms will be used interchangeably throughout, because ERBs was the term used when the research was carried out in 2016.

### Use of Soft Restraint Kits (SRK) in high secure hospitals

SRKs are used in a number of different situations, primarily to enable safe movement or transportation of a patient, or to temporarily immobilise the patient to enable treatment to be administered. SRKs are used in situations where the patient is violent, highly resistive or extremely volatile and unpredictable and where, without the use of SRKs, such interventions would present significant risks to the safety of staff, the general public or the patient.

Within the Mechanical Restraint System policy of the Scottish high secure hospital, the use of SRKs must be a reasonable and proportionate response to the risk presented by the patient. The decision to apply mechanical restraint is a clinical decision and must be underpinned by a clear clinical rationale and treatment plan that details the clinical monitoring regime, the reporting regime, and the review procedures associated with the intervention.

There are three patients currently prescribed SRKs within the Scottish high secure hospital. Governance of their use is stringent. The prescription of use is for instances of extreme prolonged violence, serious self-harm and for treatment or medical attention, obtain blood samples and the relocation in the event of a fire evacuation. Their use requires approval from two Directors from the Senior Management Team and from the Mental Welfare Commission (an external governing body). Prior to the current research project the use of SRKs had been brief and limited to the transportation for medical intervention to a general hospital; they had never been used for prolonged periods such as the situation under scrutiny. This was quite a unique situation, worthy of evaluation.

### Effects on the Nursing Role and Relationship

Although ward based nurses within forensic mental health, settings have many important roles, including maintaining a safe and secure environment, a priority is to establish the caring nurse-patient relationship (14). It is this priority that creates the difficulty when the patient is in seclusion, extended time-out or using SRKs. Patients who display significant challenging or dangerous behaviours may require a tailored care plan enabling them to be cared for in isolation from their peers. In these exceptional circumstances, there may be a need to modify their environment and at times have their own nursing care team, separate from the ward. In the most extreme cases, seclusion and/or extended time-out may last for prolonged periods of the day, sometimes months and years.

The role of a nurse and the relationship and interactions between a nurse and patient within forensic psychiatric care has received increased interest over the past few decades (15–19). Some of the literature associated with seclusion reflects a negative view of caring for in-patients within psychiatric care. The focus is predominantly on decision-making and management procedures (20, 21), nurses and patients attitudes (22–24) and the satisfaction of service users expectations of mental health care (25, 26). There seems to be a dearth of literature exploring the role and relationship of the nurse and the extreme cases of patients detained within high secure forensic services with tailored nursing care plans when cared for in seclusion and self-isolation including patients using SRK’s (27). A more recent review suggests, that many clinicians support the use of seclusion as a safe, and even therapeutic, intervention (28). Perhaps these positive attitudes towards seclusion would diminish if it was used less frequently.

### Aim

The aim of this study is to explore the perceptions of staff nursing a patient in Soft Restraint Kits over a sustained period of time.

### Methodology

In order to achieve the aim and address the research questions a qualitative method was adopted. The greatest strength of qualitative design is that it enables the researcher to study phenomena which would otherwise be unachievable (29). It also allows the researcher to acquire a more in-depth understanding of the phenomena (30) and enables participant’s thoughts, feelings and experiences to be heard (31). As this study sought to explore participant’s perceptions which are likely to include thoughts feelings and experiences directly relating to the use of SRKs, then this design was deemed suitable.

## Method

### Case study

This article is based on the experience of managing one particular case, personal details are restricted in order to maintain anonymity. Mr E was admitted to the high secure hospital following a serious offence. He presented with florid psychotic symptoms, was unresponsive to medication and was unmanageable within the main ward. The level of violence towards himself and others escalated over a period of weeks and was so extreme and of sufficient intensity that a decision was made by the Senior Management Team to use mechanical restraint - SRK – as part of his ongoing care and treatment programme. The patient was isolated from the main ward and nursed in the Modified Strong Room (MSR), more commonly used for seclusion. Initially three belts were used with soft cuffs, one on the chest, abdomen and knees. The patient was lying in a supine position on the floor. The belts were on twenty four hours a day. Gradually, one by one the belts were loosened then removed entirely.

Research questions

- What is the role of the nurse in caring for patients using SRK’s?
- What are the factors that influence and inhibit the role of the nurse in caring for patients using SRK’s?
- What skills are required to assess, plan and record interventions related to caring for patients using SRK’s?

### Sample

A purposive sample of ten registered and unregistered female and male staff drawn from wards across the high secure hospital were selected for interview by the Principal Investigator (PI); the PI was a Consultant Nurse who had worked in forensic services for nearly twenty years. Demographic details of the sample were limited once again to protect the anonymity of participants. Braun and Clarke (32) maintain that sample size of 15 – 30 participants is typical of a qualitative study; however these numbers can vary depending on what information is being sought. This size of sample is perhaps small but acceptable for the purpose of gathering useful information on this specialist subject, and did include 50% of the staff trained in the use of SRKs at the time. The sample was identified through discussion with Lead Nurses within the hospital. This particular group had been directly involved in the care and treatment of the patient during the two month period the patient was restrained using SRKs and were thus in an ideal position to reflect on their experience. They were all very experience members of staff, having worked in the hospital for many years; part of a small group of twenty who had received specialist (one day) training in the use of ERBs prior to implementation.

### Interview

Rather than use structured interview whereby the information gathered could have been restricted, semi-structured interviews were undertaken allowing participants the flexibility to explain their own experiences in detail without being restricted to a tight framework (33). The main questions forming the basis of the interview guide were: 1) what are the skills and roles of the nurse? 2) is there anything that inhibits/enhances the way that you work? 3) what qualities are required to be able to work with people in ERBs? 4) what do you think about the training that was provided? 5) how would you describe the nurse patient relationship in these circumstances?

### Ethical considerations

All participants were approached by the PI and given an information leaflet offering details of the study. They were advised they had seven days to consider their involvement, but were under no obligation to do so. Following the seven day period they were re-approached by the PI and asked to sign a consent form prior to participation. Participants were assured that as far as possible their anonymity would be preserved when reporting. All participants were issued a unique identifier (P1–P10). The confidential data they provided was stored in a locked cabinet in the PIs office, where only researchers have access. Due to changes in legislation in 2012 in the United Kingdom staff interviews no longer require ethical approval from the Integrated Research Application Service (IRAS). The proposal was still subject to scrutiny through the local Research Committee.

### Data analysis

Thematic analysis (TA) was used to analyse the data generated from the semi-structured interviews carried out by the first author. TA was selected over Interpretive Phenomenological Analysis (IPA) for pragmatic reasons. There were ten people available for interview (n=10) and the interviews were likely to be shorter using TA, thus less time would be used for transcription; this was part of a wider study which had a restricted time-frame. Thematic analysis is a method for identifying and interpreting themes from the data (34). The advantages of this type of analysis are that it is a flexible and useful research tool, which can provide a rich and detailed account of the data (35). Analysis begins with reading and familiarising of the data. Initial codes are then made which involved organising the data into meaningful groups. The data is then read and reread with the aim of identifying repeated patterns and how different codes may combine to form a theme. The data analysis includes multiple levels of interpretation to detect inconsistencies, contradictions and researcher bias (35). Finally, once the themes are generated the validity of the themes in relation to the data set are considered. This ensures that the themes reflect an accurate representation of the data that is transparent to the reader.

### Rigour

Both authors were from a nursing background and had significant research experience, thus had credibility with the participants because they were able to relate to them. The first author no longer works in the organisation so there was no personal connection and this prevented any perceived and undue pressure on participants, in addition to reducing the chance of biased responses. Interviews were undertaken, transcribed and analysed by the co-authors. Following transcription the scripts were returned to each participant to check for accuracy. This process of respondent validation is well recognised as a strategy used to ensure the strength and credibility of the research (36, 37). Both researchers (HW and LT) reviewed each transcript against their original audio file. The content of the interviews was compared and contrasted in order to ensure saturation had been reached. The views expressed represent a comprehensive review of staff experiences. The underlying patterns and notions that emerged into themes were based on the reflections of the co-authors and quotes to match each theme were selected by mutual agreement.

## Results

Four major themes were identified from the transcripts: sense of responsibility, aptitude, inhibitors and enablers and consequence. All of these themes (in bold) were further subdivided based on the analysis (in italics) see **Box 1** for a summary of all themes.

Box 1 **Summary of super-ordinate and sub-ordinate themes emerging from interviews.**

**Table:** 

**Theme**	**Sub-theme**
**Sense of responsibility**	*Changes over time* *Compassion* *Patient Safety* *Staff Safety* *Dignity* *Maintaining hope*
**Aptitude**	*Observation skills – recording/monitoring* *Physical skills - attending to needs* *Relational skills - maintaining therapeutic relationship*
**Inhibitors and Enablers**	*Restricted movement* *Acts of violence* *Patient’s Mental State* *Training* *Feeling valued*
**Consequence**	*Withdrawal* *Intensity* *Pressure* *Anxiety* *Burnout*

### Sense of Responsibility

There was an overwhelming ‘Sense of Responsibility’ voice by the nurses, making this the first super-ordinate theme. The strength of emotion staff felt was palpable and this clearly affected the role they assumed. The tensions between what they thought they ought to do as a nurse and what was being asked of them in this dynamic situation was evident. Their thoughts were firstly for the patient and secondly for themselves and this sense of professionalism was admirable. Sub-ordinate themes included *changes over time, compassion, patient safety, staff safety and dignity*.

#### Changes Over Time

In relation to the role and the skills used, there was a suggestion that the role changes over time.

‘It changes minute to minute, day to day, you have to adapt your role to meet the patient’s needs at that time’ (P2).

For some participants changes over time intimated a more positive attitude towards the ERBs.

‘Initially I was taken aback a wee bit, the patient was cuffed up and strapped, lying down on the ground and I thought ….how did we get to this stage? How are we going to feed him and get him to the toilet? Once we got used to the process it seemed easier as time went on ….I did wonder if he was ever going to get better…. there is a kind of release on the nursing staff though because once you get someone into the belts there’s less chance that the patient can assault them, so they don’t feel under threat anymore – but on the other side the patient’s anxiety increases’ (P10).

Not everyone shared this view, one participant was more reticent.

‘I’m not sure if peoples’ attitudes do change over time, people seem to get their head around it and think it can be acceptable in the short term, but it’s definitely not ideal for long term use……the waters are muddied a bit on this one’ (P1).

#### Compassion

Compassion was expressed when reflecting on the situation under which the patient was being nursed.

‘It had to be done, although it was not a pleasant experience, it’s not nice, I felt terrible having to do it. I felt bad because I’ve never seen a patient so stressed….it was kind of sad, when it gets to that stage that someone’s mental health has deteriorate so much that they are so violent, but they are in total crisis, that this is what it’s come to – that you’re having to use mechanical restraints’(P4).

#### Patient Safety

Patient Safety was at the forefront of participants’ minds and a pragmatic focus was evident.

‘Right from the start when you’re putting them (the belts) on, you are making sure they are safe and secure … they (the patient) are very dependent on you … if you’re not there they’re going to injure themselves’(P1).

‘I think it’s very effective….the fact that you’re keeping the patient safe is good, there’s nothing worse than seeing somebody mentally unwell slapping themselves off walls and things like that, so the fact you’re keeping them safe is good’….it’s very hard to beat’ (P2).

Others questioned the approach to patient safety and wondered if something else could be done.

‘I know the reasons why we’re having to use it, but it still goes against the grain of what you’ve been taught, I’m talking about de-escalation, least restrictive and this is at the end really’ (P4).

#### Staff Safety

Staff safety was also highlighted and participants commented on how the use of ERBs reduced the risk for everyone involved.

‘From a purely security point of view and keeping the staff and other patients safe then it’s good, but not ideal … it’s like a necessary evil’ (P1).

‘It allows us to administer treatment safely, to protect the patient and other people. I do think there are lots of benefits from the ERBs as long as it is safely governed, that is vital … they have worked wonders for some of our patients…. I’ve only ever seen them used in extreme cases where there was absolutely no other way we would have been able to administer medicine….I wouldn’t like to see them used regularly; it worked for this patient – thankfully- it allowed him to get medicated and recover’ (P3).

#### Dignity

The issue of dignity evoked a strong response and divided opinion, around half the participants felt the ERBs helped maintain dignity.

‘When used for emergency treatment it preserves patient’s dignity because they can be put in a wheelchair with a blanket over them (when taken to a general hospital)’, this maybe reduces the stigma rather than in handcuffs or holds with three members of staff (P3).

‘I think it’s more dignified, it’s less restrictive and it does give patient that space, so you don’t need three people on top of the person….in that respect it’s more acceptable’ (P6).

The other half were more dismissive, commenting on the fact it could be degrading and limits independence.

‘From a nursing point of view I’m not sure it’s as dignified as it could be for the patients, if I’m honest…. the patient is totally powerless at the end of the day’ (P1).

### Maintaining Hope

The resilience demonstrated by staff was remarkable and it seemed to be drawn from their need to maintain hope.

‘You’d come in each day and count the belts and sometimes your heart would just sink because there was one more than yesterday, but it was great when it worked the other way round’ (P5).

‘Amidst torrid abuse ‘there were spells of times when the patient was humorous and showed a likeable side to him…… that kept us going’(P6).

#### Aptitude

The wider role of the nurse was captured under the second super-ordinate theme ‘Aptitude’. This is where the competence of nurses emerged and both practical and relational elements were highlighted. Sub-ordinate themes included observation skills, physical skills and relational skills.

### Observation Skills

All staff were of the impression that monitoring was done in keeping with the policy, yet their reporting on the frequency of checks was variable.

‘Checks need to be done a minimum of 4 hourly, but you would be constantly checking, when applied the belts are checked for tightness anyway but you would check to see they (the patient) are breathing ok’. ‘You also need to check the colour in their fingers’. Routine monitoring of the physical stuff is done. There should be an up to date care plan that includes why we’ve put them on, what the objectives are and we need to make sure that it is reviewed regularly’(P3).

There also seemed to be inconsistency in views relating to recording and reporting.

‘There’s nothing formal in regard to what you put in the nursing report, unless just a brief sum up of the checks and stuff you have been doing. Normally we would just write about how the patient’s been and present a story of what’s happened in the last 2 hours or so - notes were entered into the electronic patient recording system RIO….a bit of structure would be good so that things don’t get missed’(P4).

‘We put a formal process in place for recording as we didn’t have a proper policy, because we hadn’t used the process before and we thought it better to record too much rather than not enough’ ‘physical observations had to be carried out and recorded, they were checked, I think every 2 or 4 hours. I think we were a bit cautious and we maybe didn’t need to do the observations quite as much’(P6).

### Physical Skills

Attending to physical needs was much more of an issue than participants originally anticipated, and it created quite a strength of feeing.

‘We did what we had to do – it was like caring for the elderly….we had to do everything for him, feed him toilet him, wash him – there’s no dignity in that’ (P10).

‘Because you were feeding him, giving him fluids, cleaning him and so on, that in itself puts a certain amount of dependency on you. He was defecating and urinating in the belts deliberately – that was behavioural – it seemed to be the only way he could get back at us…… what else could he do’ (P6).

### Relational Skills

There were mixed views in relation to people’s ability to develop and maintain a therapeutic relationship with the patient. One or two participants were quite adamant that…

‘It’s much easier to nurse people in ERBs when you know them’ (P7).’The opportunity to develop a therapeutic relationship was there because you were spending so much time with him; it didn’t seem much different to working with any other patient. I don’t think the ERBs changed it in any way, shape or form at all, the good thing was that the guy was seeing the same people everyday….but from a personal view you just get on with it’ (P6).

Normalising the experience was important to staff.

‘You need to make sure you are still treating them (the patient) as you normally would, whether nine times out of ten they are there because they have been assaultive, you need to make sure you’re not being judgemental, that you are trying to maintain a normal relationship with them’ (P4).

Others thought perhaps the patients understanding of why ERBs were applied might have affected the relationship.

‘Because his behaviour was mostly mental health driven at least we still had a bit of a good relationship with the guy and as his mental health started to improve the relationship started to build itself back up. There was no him and us sort of thing, it was just a case of keep going to get through to him’ (P2).

Arguably, close proximity helps to maintain a therapeutic relationship

‘You could say it didn’t hinder the relationship because you were with one person instead of 12 in a ward, so you’re closer with him and engaging with him’ (P2).

#### Inhibitors and Enablers

The third theme outlines some of the more positive and negative aspects of working with patients in ERBs, entitled: Inhibitors and Enablers’; This was broken down into five sub-ordinate themes—the first three were inhibitors and the last two enablers: restricted movement, acts of violence, patient’s mental state, training, and feeling valued.

There were clear challenges due to the circumstances and things that got in the way of good practice, these were identified as **‘Inhibitors’**.

### Restricted Movement

Staff struggled with the restrictions of the environment.

‘It’s hard though because of the position he’s in, belted up and you have very limited movement, they don’t want to engage with you … when dealing with this guy he just wanted to get out so he could hit you’ (P2).

‘You can’t use touch – if you know the patient well you can use a certain level of touch to reassure them, but you don’t in this situation, you kind of stand back and observe the patient – it acts like a barrier between you and the patient – physically and the way you relate……their social interactions are completely different’ (P10).

### Acts of Violence

The violence continued, despite the use of the ERBs and staff needed to react quickly on many occasions.

‘The level of aggression was so intense….at one point he was going to throw a cup of tea over me, but we were close enough that we were able to intervene quickly’(P8).

‘He tended to get angry. It was behaviours, he would describe it as ‘night dreams’, and if he woke up from a ‘night dream’ he would be frightened – that could kick the behaviour off….I think he was genuinely frightened of being killed … the behaviour would go on for a long time. I think he was happy for the staff to be in close proximity still having to hold him. Even though he was in the mechanical restraint the staff still had to hold his arms to prevent him thrashing himself about, he could still do himself a lot of damage if not held. This may be why he was spitting and urinating, because of his frustrations linked to us preventing him hurting himself’(P6).

### Patient’s Mental State

The struggle to comprehend what was driving the behaviours was unclear and staff wanted to believe it was due to the patient’s mental state rather than it being deliberate and calculated.

‘He was so unwell it was hard to know what he was taking in and he needed a lot of reassurance to make him believe we were actually trying to help him….his mental state was very poor, he had a fixed belief that he was here to be harmed and to be murdered, he seemed to be fighting for his life……’ (P10)

Participants were able to identify things that helped the role, identified here as **‘Enablers’;** also see **Box 2**.

Box 2 **Qualities of the forensic nurse identified by ward staff involved in using ERBs/SRKs.**

‘You need a ‘**long attention span**’….you’re stuck there and you can’t go there or get anything if there’s only two of you trained in ERBs, you can’t even get to the toilet.

‘You’ve got to be ‘**thick skinned**’ I think you’ve got to be like that to work in this type of area anyway because the abuse is going to come at you regardless of the ERBs. You deal with that kind of stuff in clinical supervision’ (P1).

‘You can’t be a shrinking violet and you’ve **got to be able to take the abuse and not personalise it**. I certainly wouldn’t put a new staff nurse into that situation ….I think it would finish a lot of people off. I think you’re looking for **experienced** people that have been here a relative length of time so they are able to adapt to situations’ (P7).

‘You tend to be working with a high profile patient and you are more open to scrutiny, that’s why it’s better to be experienced, ‘**be good at building rapport** quite quickly with a patient’ (P6).

‘You really need to be **resilient**’ (P5).

‘**Be tolerant** – especially when you are repeatedly being insulted, the insults were quite bad (sexually explicit, racist) which seemed deliberate. If bored he’d comment on something that would maybe get a reaction, for example, something homophobic, although staff tried not to react, if they did, he’d continue to press the buttons; **you need to know when you need to take a break**’ (P10).

‘Be very patient, understanding and be able to relate to the patient’ (P9).

‘Be **calm, objective**, feel confident in the job they are doing … people have to **feel confident using the equipment** and they have to feel supported … they also have to be effective communicators and be very observant and have **good negotiating skills**’. When people are put into a situation where they use something that they don’t use all the time, that can be very anxiety provoking for them. I think the adrenalin could be surging and sometimes it’s trying to get things done as fast as you can, possibly just because you want done….that’s where others can help and can slow it down a bit, you’re not just working on your own, **it’s about being part of a team and being a team player**’ (P8).

### Training

Staff were provided with one day training in the use of ERBs.

‘It was important that they knew about the governance of their use, the legislation and when it was appropriate to remove them immediately if there were physical problems. They also needed an underpinning knowledge of the risk factors. Staff needed to know what was expected of them whilst nursing a patient in ERBs, and the associated benefits of their use. They need to know they should never work outwith their capabilities, and they should never make decisions alone – they are a team’ (P3).

Training did prepare you.

‘The one day training is great, you feel really confident at the end of the day. If you are using ERBs regularly, but if you have one day training 7 or 8 months before you use it then that’s different. When you’re doing the training you are having the ERBs applied to yourself and it gives you a sense of how it feels, it does really feel weird and it doesn’t feel good to be honest, but it gives you an idea of what it’s like’ (P4).

Some reported feeling less ready.

‘I didn’t feel adequately prepared or confident about the task being asked’’ Felt shocked, worried, what if something goes wrong?, what are the repercussions if something bad happens?’ (P9).

### Feeling Valued

The whole process was protracted, extending across a few months. Staff simply wanted their continued efforts to be acknowledged, because of the huge amount of pressure they were under to get this right; given it was the first use of ERBs for an extended period.

‘You really want support from your line managers as well … you want some recognition of you know … you are coming in here day in day out, dealing with a really challenging patient, you’re using equipment that is predominantly unfamiliar to you, you’re being exposed to a whole array of stuff, verbal abuse, potential assaults, patients trying to self harm which can be quite distressing. So I think the recognition for the staff would be good and an acknowledgement of the difficulties people face’(P8).

#### Consequence

The fourth and final theme was ‘Consequence’. In any new situation where there is a test of change there is chance there will be a mixed response. Nursing assistants seemed very able to cope and shut off from the abuse, perhaps this is due to the fact they are with patients most of their working shift. There was a tendency for more registered staff withdrawing.

### Withdrawal

Involvement in the use of ERBs was voluntary initially and despite wanting to continue a small number dropped out of the delivery team.

‘Reasons for withdrawal – not doing what was agreed at the outset, ‘veering from the agreed plan’, rules and methods seemed to be changing and it was frightening’ (P4).

### Intensity

The staff who were on the ward where the patient was in ERBs were more likely to be involved regularly and felt the brunt of the intervention. There was an acknowledgement from all participants that it could be difficult and perhaps challenging if it was regular practice.

‘It was hard physically, because at first you were bent over and on your knees most of the shift and it becomes really uncomfortable. The patient is very dependent on you, you can’t leave their side, you are there for total care. You’re knackered at the end of a shift, cause it can be really tense, if he’s unsettled and you’re medicating then restraining, by the end of the night shift you just want to go home’ (P1).

#### Pressure

Pressure affected some more than others:

‘There’s a lot of pressure on you because we feel that this guy … the state that this guy is in he could actually die in these belts quite easy because they are quite restrictive and that’s what they’re designed for.to cut down the amount of injuries to him and us. You would go home physically knackered and mentally drained…. it’s harder than the usual work’ (P2).

‘You need to be able to soak up the pressure a wee bit in these situations … you feel a sense of responsibility to do it too…. the reason I did it was because I was the key worker for the patient and I didn’t want to let my patient down. It didn’t really get to me’ (P7).

#### Anxiety

Anxiety was evident amongst some but not all participants.

‘You never quite know if the ERB’s are going to work and everything’s going to be quite safe when patients are in them – which you would kind of anticipate – but people are still getting hurt whilst they are in them so you’re constantly on edge’ (P3).

‘I wasn’t that anxious, but I could understand people being a bit anxious because of the threat’ (P6).

#### Burnout

Burnout…. was it inevitable?

‘It could very easily be an area where staff cold burnout … there’s a high risk of that….I know there are people who have been involved then asked to get moved away and then decided not to update their training……it was because they found it very difficult’ (P6).

‘There was a horrible feeling of dread coming into work in the morning…. I avoided answering the phone at night … I didn’t want another shift’ (P9).

## Discussion

Despite the controversy over the use of seclusion and restraint, they are commonly used to treat and manage disruptive and violent behaviour (5). The more limited literature on the subject of mechanical restraints (27) makes it quite difficult to generate comparisons with findings from this current study. There certainly seems to be a move towards reducing the use of seclusion and restraint (7, 38, 39).

One large scale quantitative Australian study, reported on 512 nursing perceptions of reducing containment methods such as seclusion and mechanical restraint (9). A number of questionnaires were used to elicit responses and results do resonate with the findings from our small sample, especially in relation to nurses using their clinical skills to maintain safety.

This report focuses on a relatively unique situation for nursing staff where individual experiences were quite discrete and varied. The role of the nurse has been highlighted and many examples offered. Staff all understood the rationale for use of the SRKs and were willing participants, because they realised this was the safest option for that particular patient at that point in time. One body of literature suggests that the more professionals are personally involved in these processes the more positive they evaluate them (40, 41) and we would tend to agree with this based on the feedback from our sample; despite the difficulties incurred along the way. The necessity of restraint and use of various containment methods is supported in the context of dangerous situations, albeit as a last resort to protect both patients and staff alike (42). There is evidence to support the use of SRKs here, some staff attitudes changed over time becoming less anxious and more accepting as time progressed, however, this does not apply to everyone.

Factors that influence and inhibit the role centred around the importance of being prepared through training and feeling valued by management. Benefits of training and preparedness generally reduce anxiety even in highly tense situations such as this, indeed it should be considered an essential component of any new initiative. Through brief discussion with one member of Senior Management, from their perspective, they felt they were wholly supportive of staff.

There still appears to be a conflict in staff views with regard to the use of mechanical restraint as a method of containment, which is also reflected in the literature. Over the past decade seclusion with or without restraint has been considered therapeutic (43, 44), others have viewed it as a control measure (45). Recent reports from Gerace and Muir-cochrane (9) consider these extreme measures to be deleterious to relationships with patients. Our sample were predominantly positive about the need for SRKs but this initial experience was clearly difficult for them on a number of different levels; they were torn on the issue of therapeutic value. For this reason a small number (two) of the original group of twenty were allowed to withdraw from participation.

It would be of real interest if there was more literature on the subject, extending beyond staff perspectives, to include patients’ views; this might enable others to benefit from the learned few. A recent example of eight forensic patients’ perspectives was reported by Askola et al. (46), in this study patients’ narratives contained different themes telling different things. The suggestion was that patient’s experiences of their treatment could potentially improve the quality of patient centred care.

The Hospital involved in this study did learn from the experience and a decision was made to make training in the use of SRKs mandatory for all registered nurses, in order to minimise and hopefully eradicate burnout. Several mentioned that it might have been easier if they could have shared the load with a wider group of people and that may have served to reduced the intensity of the experience.

Two years on the patient was functioning well and was enjoying grounds access. His parents were delighted with the outcome and praised the Senior Management Team for the extreme steps they took to care for their son, because they were struggling to see any resolution to the situation.

## Strengths/Limitations

This study offered an excellent opportunity to capture the perspectives of nurses working under extremely difficult circumstances, necessitating the use of mechanical restraints with a very disturbed and distressed patient. This was a small scale study on a single site and views of the limited number of participants may not necessarily be representative of the wider forensic nursing population. There is also the likelihood of recall bias since the study was undertaken more than six months after the staff had been actively involved in the use of SRKs. This may also account for the discrepancies in reporting on procedures for recording and reporting. If future research could capture the patients’ perspective this would be of real interest to clinicians. To repeat the research in the same organisation now that all nursing staff are trained in the use of SRKs would also be of interest.

## Conclusions

This was a small scale study undertaken to capture the views of staff nursing a patient for an extended period using SRKs. The sampling techniques, data collection and analysis selected reflect the research approach used to enhance the credibility, trustworthiness and dependability of findings. Use of SRKs as a containment method generated a mixed response, perhaps best summarised as ‘a necessary evil, but a last resort’. In some sense, the patients who are deemed to require use of SRK test the limits of therapeutic relationships and relational security. Four clear themes were generated from discussions held with nursing staff. The first related to a clear ‘sense of responsibility’, with a tendency for opinion to change over time. Staff felt compassion towards the patient empathising with the situation he was in and made every effort to maintain some level of dignity. Patient and staff safety were of paramount importance, but maintaining hope was just as crucial to the success of the care plan. The second theme related to ‘aptitude’ employed whilst caring for a patient in SRKs. The necessity for good observations skills and an aptitude to record are report accurately was highlighted. The dawning realisation that all needs had to be attended to was something that staff had perhaps given less thought to during their preparation, but the stark reality of the situation meant they had to find ways to effectively deal with each issue as it emerged. Developing and maintaining a therapeutic relationship was more difficult for some than others and the SRKs seemed to instantly provide a barrier to engagement. A number of inhibitors and enables were identified through interview, generating the third theme. The constant extremes of verbal abuse and violent behaviour presented challenges and staff had to use their clinical skills to deal with this. Yet the patient’s poor mental state, which most believed was driving the behaviour, was the thing that staff focussed on more. Training did make the role easier, but feeling valued was even more important. The final theme was ‘consequence’. It became apparent that the pressure and intensity of managing this violent patient weighed heavily on the staff creating increased anxiety and a degree of burnout. It is crucial that staff are supported and protected from the potential unwelcome impact.

### Implications for practice

Situations such as that reported here are somewhat unconventional, even for forensic mental health care, but much has been learnt from these early experiences in an NHS facility:

- the use of SRKs can offer an effective method of managing extremely violent behaviour in a relatively safe and contained manner;
- preparation and planning for SRK use is essential and until people are placed in a live situation they are quite unaware of the impact it can have;
- the training could include something on the potential psychological emotional impact on staff and brief refreshers once a year should be considered;.
- support and supervision of staff is recommended during a sustained period of practice using mechanical restraints, reflective practice groups could be particularly beneficial.

## Data Availability Statement

The datasets generated for this study are available on request to the corresponding author.

## Ethics Statement

Ethics approval was not required as per local legislation and guidelines. The patients/participants provided their written informed consent to participate in this study.

## Author Contributions

Both authors contributed to the design and delivery of the study in addition to the write up of the article.

## Funding

Funding for the project was provided by The State Hospital.

## Conflict of Interest

The authors declare that the research was conducted in the absence of any commercial or financial relationships that could be construed as a potential conflict of interest.
